# Supplementary material for: Access to essential psychotropic medicines in Addis Ababa: A cross-sectional study
Source: PLoS One. 2023 Jul 14;18(7):e0283348. doi: 10.1371/journal.pone.0283348 (PMC10348529; doi:10.1371/journal.pone.0283348)
Supplement: S1 File — (DOCX) [file pone.0283348.s001.docx]

Supplementary File 1: Economical grouping of sub cities in Addis Ababa, 2019

| Burden of poverty | Sub Cities | Per capita income in birr/year | Rank |
| --- | --- | --- | --- |
| Group 1:  1%-10% | Bole | 15551 | 1 |
|  | AkakiKality | 13448 | 2 |
|  | Yeka | 12146 | 4 |
| Group 2:  11%-20% | Kirkos | 12265 | 3 |
|  | Gullele | 11008 | 6 |
|  | Nefas Silk Lafto | 10264 | 7 |
|  | KolfeKeranyo | 11059 | 5 |
| Group 3: >20% | Lideta | 8448 | 8 |
|  | Arada | 8101 | 9 |
|  | Addis Ketema | 7226 | 10 |

Source: Gebre-Egziabher et al., 2015.
