# Supplementary material for: Access to essential psychotropic medicines in Addis Ababa: A cross-sectional study
Source: PLoS One. 2023 Jul 14;18(7):e0283348. doi: 10.1371/journal.pone.0283348 (PMC10348529; doi:10.1371/journal.pone.0283348)
Supplement: S2 File — (DOCX) [file pone.0283348.s002.docx]

Supplementary File 2: The Customized WHO Medicine Price Data Collection form

**Research Questions**

This research study was aimed to answer the following questions:

1. What was the availability of essential psychotropic medicines in Addis Ababa?
2. How much did patient pay for essential psychotropic medicines in Addis Ababa?
3. Are the standard treatments affordable to individuals with low income?
4. How efficient was the EPSA procurement system in procuring low priced psychotropic medicines?

**Survey Questions**

Date: ____________________

Sub City: _______________________

Name of town/village/Woreda: ____________________________________________

Name of medicine outlet (optional):_________________________________________

Medicine outlet unique survey ID (mandatory):_________________________________

Type of medicine outlet:

- Public sector facility (specify level of care below):
  - Primary care facility
  - Secondary care facility
  - Tertiary care facility
- Private sector medicine outlet

Type of price:

- Price the patient pays
- Procurement price

Type of data:

- Sample outlet
- Validation visit

Name of person(s) who provided the medicine prices & availability data: ___________________

Name/Code of data collectors: _______________________________________________

Verification

To be completed by the principal investigator at the end of the day, once data have been verified,

Signed:________________________________________Date:_________________________

| **S/N** | **A** | **B** | **C** | **D** | **E** | **F** | **G** | **H** | **I** | **J** |
| --- | --- | --- | --- | --- | --- | --- | --- | --- | --- | --- |
|  | **Generic name, dosage form & strength** | **Medicine Type** | **Brand or Product name(s)** | **Manufacturer** | **Available**  **“Yes” or**  **“No”** | **Pack size recommended** | **Pack size found** | **Price of pack found** | **Unit price (4digits)** | **Comments** |
| 1 | Alprazolam tablet, 0.5mg. | OB | Xanax, | Pfizer |  | 10x3 |  |  | per tab |  |
|  |  | LPG |  |  |  | 10x3 |  |  | per tab |  |
| 2 | Amitriptyline tablet, 25mg. | OB | Tryptizol | MSD |  | 10x10 |  |  | per tab |  |
|  |  | LPG |  |  |  | 10x10 |  |  | per tab |  |
| 3 | Carbamazepine tablet, 200mg. | OB | Tegretol | Novartis |  | 5x10 |  |  | Per tab |  |
|  |  | LPG |  |  |  | 10x10 |  |  | Per tab |  |
| 4 | Chlorpromazine Hydrochloride - 100mg - Tablet | OB | Largactil | Sanofi |  | 100x10 |  |  | Per tab |  |
|  |  | LPG |  |  |  | 100x10 |  |  | Per tab |  |
| 5 | Clomipramine capsule, 25mg | OB | Anafranil | Patheon Inc |  | 25x10 |  |  | Per cap |  |
|  |  | LPG |  |  |  | 25x10 |  |  | Per cap |  |
| 6 | Clozapine tablet, 25mg | OB | Clozaril | Novartis |  | 5x10 |  |  | Per tab |  |
|  |  | LPG |  |  |  | 10x5 |  |  | Per tab |  |
| 7 | Diazepam tablet, 5mg. | OB | Valium | Roche |  | 10x10 |  |  | Per tab |  |
|  |  | LPG |  |  |  | 10x10 |  |  | Per tab |  |
| 8 | Fluoxetine 20 mg cap. | OB | Prozac | Eli Lilly |  | 10x2 |  |  | Per cap |  |
|  |  | LPG |  |  |  | 10x2 |  |  | Per cap |  |
| 9 | Fluphenazine decanoate 25mg/ml in 1ml ampoule | OB | Prolixin | Novex Pharma |  | 10 |  |  | Per ml |  |
|  |  | LPG |  |  |  | 10 |  |  | Per ml |  |
| 10 | Haloperidol tablet, 2mg | OB | Haldol | Janssen Pharma Inc. |  | 10x10 |  |  | Per tab |  |
|  |  | LPG |  |  |  | 10x10 |  |  | Per tab |  |
| 11 | Imipramine tablet, 25mg. | OB | Tofranil | Excellium Pharma Inc. |  | 20x5 |  |  | Per tab |  |
|  |  | LPG |  |  |  | 20x5 |  |  | Per tab |  |
| 12 | Lamotrigine tablet,50mg. | OB | Lamictal | GSK |  | 10x3 |  |  | Per tab |  |
|  |  | LPG |  |  |  | 10x3 |  |  | Per tab |  |
| 13 | Lithium Carbonate tablet, 300mg. | OB | --------- | --------------------- | --------- | -------- | ----- | --- | --- | no O.B |
|  |  | LPG |  |  |  | 10x10 |  |  | Per tab |  |
| 14 | Olanzapine tablet 5mg. | OB | Zyprexa | Eli Lilly & Company |  | 10x10 |  |  | per tab |  |
|  |  | LPG |  |  |  | 10x10 |  |  | per tab |  |
| 15 | Phenobarbital tablet, 30mg | OB | Luminal | AstraZeneca |  | 100 |  |  | per tab |  |
|  |  | LPG |  |  |  | 100 |  |  | per tab |  |
| 16 | Phenytoin tablets, 100mg. | OB | Dilantin | Pfizer |  | 100 |  |  | Per tab |  |
|  |  | LPG |  |  |  | 100 |  |  | Per tab |  |
| 17 | Risperidone tablet, 1mg | OB | Risperdal | Janssen Pharma Inc. |  | 10x10 |  |  | Per tab |  |
|  |  | LPG |  |  |  | 10x10 |  |  | Per tab |  |
| 18 | Sertraline tablet, 50mg. | OB | Zoloft | Pfizer |  | 10x10 |  |  | Per tab |  |
|  |  | LPG |  |  |  | 10x10 |  |  | Per tab |  |
| 19 | Sodium Valproate tablet, 200mg | OB | Depakote, | Abbott Laboratories; |  | 10x10 |  |  | Per tab |  |
|  |  | LPG |  |  |  | 10x10 |  |  | Per tab |  |
| 20 | Trifluoperazine HCL, 1 mg tablet | OB | Stelazine | Vianex SA |  | 10x10 |  |  | Per tab |  |
|  |  | LPG |  |  |  | 10x10 |  |  | Per tab |  |
