# Supplementary material for: Access to essential psychotropic medicines in Addis Ababa: A cross-sectional study
Source: PLoS One. 2023 Jul 14;18(7):e0283348. doi: 10.1371/journal.pone.0283348 (PMC10348529; doi:10.1371/journal.pone.0283348)
Supplement: Attachment — Submitted filename: PONE-D-22-17182_reviewer report. pdf.pdf [file pone.0283348.s005.pdf]

PLOS ONE

Access to Essential Psychotropic Medicines in Addis Ababa: A Cross-Sectional Study

--Manuscript Draft--

**Manuscript Number:**	PONE-D-22-17182
**Article Type:**	Research Article
**Full Title:**	Access to Essential Psychotropic Medicines in Addis Ababa: A Cross-Sectional Study
**Short Title:**	Access to Essential Psychotropic Medicines

Corresponding Author: Teferi Gedif Fenta, PhD
School of Pharmacy, Addis Ababa Univesrity
Addis Ababa, ETHIOPIA

Keywords: Keywords: Psychotropic Medicines; Availability; Prices; Affordability; Addis Ababa; Ethiopia.

Abstract:

Access to Essential Psychotropic Medicines in Addis Ababa: A Cross-Sectional Study.

Molla Teshager Ayehu1, Teferi Gedif Fenta2*, and Mesfin Araya3

1 Department of Pharmaceutics and Social Pharmacy, School of Pharmacy, College of Health Sciences, Addis Ababa University, Addis Ababa, Ethiopia.
2 Department of Pharmaceutics and Social Pharmacy, School of Pharmacy, College of Health Sciences, Addis Ababa University, Addis Ababa, Ethiopia
3Department of Psychiatry, School of Medicine, College of Health Sciences, Addis Ababa University, Addis Ababa, Ethiopia.

*Corresponding Author: Teferi Gedif Fenta, tgedif@gmail.com

Abstract

Background: Mental disorders are becoming a growing public health problem worldwide, especially in low- and middle-income countries. Regular and adequate supplies of appropriate, safe, and affordable medications are required to provide quality mental health services. However, significant percentages of people with severe mental disorders are not getting the treatment. Among others, the availability and affordability of psychotropic medicines are significant barriers for many patients in meeting their medication needs. This study aimed to assess the availability, prices, and affordability of essential psychotropic medicines in the private and public health sectors of Addis Ababa, the capital city of Ethiopia.
Methods: A cross-sectional study design was used in 60 retail medicine outlets. Stratified random and quota sampling were applied to select the retail outlets. Data was entered and analyzed using the pre-programmed WHO/HAI workbook and SPSS V.25.
Results: The mean availability of LPG psychotropic medicines was 24.33% in Addis Ababa (28.7% in the public sector and 19.80% in the private sector). The patient prices for the LPG ranged from 0.52-6.43 MPRs in public and 1.08- 24.28 MPRs in private sectors. Standard treatment cost varied from 0.1–7.8 days' wages in public and 0.8-25 days' in private sectors for the lowest-paid government worker to purchase a month's supply.
Conclusions: Essential psychotropic medicines were poorly available with high prices and low affordability in Addis Ababa. To ensure access, an efficient supply across all levels of care and financial protection for essential medicines should be in place.

Order of Authors:

Molla Teshager, BPharm, MSc

Teferi Gedif Fenta, PhD

Mesfin Araya, MD, PhD

Additional Information:

Question	Response
**Financial Disclosure**<br><br>Enter a financial disclosure statement that describes the sources of funding for the work included in this submission. Review the submission guidelines for detailed requirements. View published research articles from *PLOS ONE* for specific examples.<br><br>This statement is required for submission and **will appear in the published article** if the submission is accepted. Please make sure it is accurate.<br><br>**Unfunded studies**<br>Enter: *The author(s) received no specific funding for this work.*<br><br>**Funded studies**<br>Enter a statement with the following details:<br>• Initials of the authors who received each award<br>• Grant numbers awarded to each author<br>• The full name of each funder<br>• URL of each funder website<br>• Did the sponsors or funders play any role in the study design, data collection and analysis, decision to publish, or preparation of the manuscript?<br>• **NO** - Include this sentence at the end of your statement: *The funders had no role in study design, data collection and analysis, decision to publish, or preparation of the manuscript.*<br>• **YES** - Specify the role(s) played.<br><br>* typeset	The Authors received no specific funding for this work
**Competing Interests**<br><br>Use the instructions below to enter a competing interest statement for this submission. On behalf of all authors, disclose any competing interests that could be perceived to bias this work—acknowledging all financial support and any other relevant financial or non-financial competing interests.	The authors declared that no competing interests exists

This statement is required for submission and **will appear in the published article** if the submission is accepted. Please make sure it is accurate and that any funding sources listed in your Funding Information later in the submission form are also declared in your Financial Disclosure statement.<br><br>View published research articles from *PLOS ONE* for specific examples.<br><br>**NO authors have competing interests**<br><br>Enter: *The authors have declared that no competing interests exist.*<br><br>**Authors with competing interests**<br><br>Enter competing interest details beginning with this statement:<br><br>*I have read the journal's policy and the authors of this manuscript have the following competing interests: [insert competing interests here]*<br><br>* typeset	
**Ethics Statement**<br><br>Enter an ethics statement for this submission. This statement is required if the study involved:<br><br>• Human participants<br>• Human specimens or tissue<br>• Vertebrate animals or cephalopods<br>• Vertebrate embryos or tissues<br>• Field research<br><br>Write "N/A" if the submission does not require an ethics statement.<br><br>General guidance is provided below. Consult the submission guidelines for detailed instructions. **Make sure that all information entered here is included in the Methods section of the manuscript.**	N/A

**Format for specific study types**<br><br>**Human Subject Research (involving human participants and/or tissue)**<br>• Give the name of the institutional review board or ethics committee that approved the study<br>• Include the approval number and/or a statement indicating approval of this research<br>• Indicate the form of consent obtained (written/oral) or the reason that consent was not obtained (e.g. the data were analyzed anonymously)<br><br>**Animal Research (involving vertebrate animals, embryos or tissues)**<br>• Provide the name of the Institutional Animal Care and Use Committee (IACUC) or other relevant ethics board that reviewed the study protocol, and indicate whether they approved this research or granted a formal waiver of ethical approval<br>• Include an approval number if one was obtained<br>• If the study involved *non-human primates*, add *additional details* about animal welfare and steps taken to ameliorate suffering<br>• If anesthesia, euthanasia, or any kind of animal sacrifice is part of the study, include briefly which substances and/or methods were applied<br><br>**Field Research**<br><br>Include the following details if this study involves the collection of plant, animal, or other materials from a natural setting:<br>• Field permit number<br>• Name of the institution or relevant body that granted permission	
**Data Availability**<br><br>Authors are required to make all data underlying the findings described fully available, without restriction, and from the time of publication. PLOS allows rare exceptions to address legal and ethical concerns. See the PLOS Data Policy and FAQ for detailed information.	Yes - all data are fully available without restriction

Powered by Editorial Manager® and ProduXion Manager® from Aries Systems Corporation

A Data Availability Statement describing where the data can be found is required at submission. Your answers to this question constitute the Data Availability Statement and **will be published in the article**, if accepted.<br><br>**Important:** Stating 'data available on request from the author' is not sufficient. If your data are only available upon request, select 'No' for the first question and explain your exceptional situation in the text box.<br><br>Do the authors confirm that all data underlying the findings described in their manuscript are fully available without restriction?	
**Describe where the data may be found in full sentences. If you are copying our sample text, replace any instances of XXX with the appropriate details.**<br><br>• If the data are **held or will be held in a public repository**, include URLs, accession numbers or DOIs. If this information will only be available after acceptance, indicate this by ticking the box below. For example: *All XXX files are available from the XXX database (accession number(s) XXX, XXX.*).<br>• If the data are all contained **within the manuscript and/or Supporting Information files**, enter the following: *All relevant data are within the manuscript and its Supporting Information files.*<br>• If neither of these applies but you are able to provide **details of access elsewhere**, with or without limitations, please do so. For example:<br><br>*Data cannot be shared publicly because of [XXX]. Data are available from the XXX Institutional Data Access / Ethics Committee (contact via XXX) for researchers who meet the criteria for access to confidential data.*<br><br>*The data underlying the results presented in the study are available*	The data underlying the results is available from the first Author

from (include the name of the third party and contact information or URL).<br>• This text is appropriate if the data are owned by a third party and authors do not have permission to share the data.<br><br>* typeset	
Additional data availability information:	Tick here if your circumstances are not covered by the questions above and you need the journal’s help to make your data available.

Powered by Editorial Manager® and ProduXion Manager® from Aries Systems Corporation

Manuscript Click here to access/download;Manuscript;Psy MS-1.docx

Access to Essential Psychotropic Medicines in Addis Ababa: A Cross-Sectional Study.

Molla Teshager Ayehu[1], Teferi Gedif Fenta[2]*, and Mesfin Araya[3]

[1] Department of Pharmaceutics and Social Pharmacy, School of Pharmacy, College of Health Sciences, Addis Ababa University, Addis Ababa, Ethiopia.

[2] Department of Pharmaceutics and Social Pharmacy, School of Pharmacy, College of Health Sciences, Addis Ababa University, Addis Ababa, Ethiopia

[3]Department of Psychiatry, School of Medicine, College of Health Sciences, Addis Ababa University, Addis Ababa, Ethiopia.

*Corresponding Author: Teferi Gedif Fenta, tgedif@gmail.com

Abstract

Background: Mental disorders are becoming a growing public health problem worldwide, especially in low- and middle-income countries. Regular and adequate supplies of appropriate, safe, and affordable medications are required to provide quality mental health services. However, significant percentages of people with severe mental disorders are not getting the treatment. Among others, the availability and affordability of psychotropic medicines are significant barriers for many patients in meeting their medication needs. This study aimed to assess the availability, prices, and affordability of essential psychotropic medicines in the private and public health sectors of Addis Ababa, the capital city of Ethiopia.

Methods: A cross-sectional study design was used in 60 retail medicine outlets. Stratified random and quota sampling were applied to select the retail outlets. Data was entered and analyzed using the pre-programmed WHO/HAI workbook and SPSS V.25.

Results: The mean availability of LPG psychotropic medicines was 24.33% in Addis Ababa (28.7% in the public sector and 19.80% in the private sector). The patient prices for the LPG ranged from 0.52-6.43 MPRs in public and 1.08- 24.28 MPRs in private sectors. Standard treatment cost varied from 0.1–7.8 days' wages in public and 0.8-25 days' in private sectors for the lowest-paid government worker to purchase a month's supply.

Conclusions: Essential psychotropic medicines were poorly available with high prices and low affordability in Addis Ababa. To ensure access, an efficient supply across all levels of care and financial protection for essential medicines should be in place.

Keywords: Psychotropic Medicines, Availability, Prices, Affordability, Addis Ababa, Ethiopia.

Introduction

Mental health matters most for the well-being of every one of us. Moreover, excellent mental health is a prerequisite for the well-being of the individuals and the societies and the country at large (1, 2). Despite these facts, large numbers of people are affected by severe mental disorders worldwide (3). ~~For instance,~~ mental illness is the leading cause of non-communicable disorders in Ethiopia. ~~It~~ accounts for 11% of the total burden of diseases in the country, and about 8000 people commit suicide every year (4). Also, evidence from cohort studies shows that persons with severe mental health conditions have died about 30 years earlier than that of the general population in Ethiopia (6). Moreover, despite the increased demand for mental healthcare services, the treatment gap is as high as 90% (7), while there are medicines in the market for most mental illnesses (8, 9). Several effective medicines are available for mental disorders, but not all "effective" drug therapies are essential (10).

Essential psychotropic medicines are those medicines that satisfy the priority mental health care needs of the population. These medicines should be made available at all levels of health care: continuously, in adequate amounts, in the appropriate dosage forms, with assured quality and adequate information, and at a price the individual and the community can afford (11). Access to essential psychotropic medicines is part of a fundamental human right (12, 13), and ensuring access to medicines for mental disorders can be beneficial not only for the patients themselves but also for employers, through reduced absenteeism and higher productivity; for family members and friends, lowering the burden of care; and for government, through reduced social security benefits (14, 15). However, compared to other essential medicines generally, the availability and affordability of medicines for mental disorders are even worse for the populations of low-income countries where mental illnesses are the enormous causes of morbidity, disability, and mortality (1, 16, 15). As a result, lack of access to essential psychotropic medicines can significantly contribute to the public health burden of untreated mental disorders (18).

In Ethiopia, the number of patients seeking care for a wide range of mental illnesses has grown from time to time and has also increased the need for psychotropic medications (4, 5, 19). However, in our setting,

most of the resources (staff, budgets, and beds) for mental healthcare services are located in Addis Ababa City, where the only mental hospital in the country is also found that serves the whole nation as the highest referral and training center in mental health (5). Moreover, the finding of studies confirmed that the prevalence of the common mental disorders in Addis Ababa is higher than the national prevalence rate (6, 20). This might be attributed to the nature of urbanization and lifestyle changes in the city (1, 21). Having this in mind, ensuring access to basic psychotropic medicines has been considered essential for the mental healthcare services provided to patients in the city (1). ~~However,~~ accessibility of treatments for mental disorders is relatively low for numerous reasons despite the considerable burden of mental illness globally (8, 22).

As worldwide evidence showed that access to essential medicines could be mainly affected by stock-out, high prices, and unaffordability, amongst others (23). Especially in the public sector, availability is relatively low. This, in turn, results in medicines being purchased with higher out-of-pocket expenses from private medicine outlets even though securing accessibility of essential psychotropic medicines in both sectors is equally crucial (19, 22[illegible]imilarly, in Ethiopia, evidence indicates that due to inadequate budget, centralization of the mental healthcare services, out-of-pocket payments, lack of human resources, and other factors make medicines for mental illnesses permanently unavailable even if selected psychotropic drugs from the different groups have been included in the national list for essential medicines. Furthermore, most mental health seekers remain under-treated (24, 9, 25). Consequently, it increases the risk of relapse, re-hospitalization, comorbidities, and premature death (2014[illegible]

~~Likewise, in Addis Ababa, the problem of access to psychotropic medicines is likely to be a matter of concern for mental health seeker~~[illegible]owever, the issue of accessibility to essential psychotropic medicines in Addis Ababa city is not well studied. At the local and national levels, few studies have examined the availability, price, and affordability of essential medicines in general. However, studies examining psychotropic medicines' availability, prices, and affordability are minim[illegible]s a result, policy-makers find it challenging to set priority concerns and testing of implementation in improving access to treatments for

mental disorders (26). Above all, any further efforts to expand access to mental healthcare services can be fruitless without ensuring the sustainable availability and affordability of essential psychotropic medicines (27). Thus, this study aims to measure the availability, price, and affordability of essential psychotropic medicines at public and private medicine retail sectors in Addis Ababa, where mental health referral hospitals for the whole country are located.

Methods

An institution-based cross-sectional study was carried out between 30 July and 18 September 2019 using WHO/HAI tools to collect price and availability from pubic and private sectors in Addis Ababa, Ethiopia's capital city. Addis Ababa has a population size of more than 3.8 million (28). During the study period, there were 1919 health facilities in Addis Ababa; of which 110 of them were public healthcare facilities and 558 were private retail pharmacies.

The study population was 668 medicines retail outlets from which the sampled outlets had been selected using combinations of stratified and purposive samplings. The sample size was determined based on the WHO/HAI manual (29). Accordingly, 60 retail outlets; 30 each from the public and private sectors, were included.

Selection of the Medicines to be surveyed

Despite the variations in their dosage forms and strengths, there are 26 essential generic psychotropic medicines included in the national medicine list of Ethiopia (30). Initially, the selection of these medicines specific to dosage forms and strengths has been made in light of the WHO Model List of Essential Medicines, the National Essential Medicine Lists of Ethiopia (30, 31), along with expert opinion from Addis Ababa University and feedbacks from Health Action International. Although 26 psychotropic medicines with specific strength and dosage forms have been used for the pilot test, six medicines were not found in any piloted retail outlets. The reasons are that 4 of the six medicines are used only as emergency medicines at the inpatient level and not stocked in most outpatient pharmacies, where their availability is less important for outpatient users. These medicines included: clonazepam 1mg/ml

injection, magnesium ~~SO4~~ 50% in 20ml injection, lorazepam 1mg/ml injection, midazolam HCL 1ml/ml injection. The other two, namely; bromazepam 3mg and quetiapine 50mg tablets, do not have suppliers in Ethiopia. Besides, bromazepam 3mg and quetiapine prices were not found in the MSH-2015 price indicator. So, the six medicines were excluded from the survey lis[illegible]herefore, the total number of medicines included in the final study was 20 essential psychotropic medicines. For each medicine in the survey, up to two products were considered: the originator brand (the original patented pharmaceutical product) and the lowest-priced generic equivalent (LPG). The general categories of medications were anxiolytics, antipsychotics, antidepressants, antiepileptic, and mood stabilizers

Eligibility Criteria of the Medicine Outlets

Inclusion Criteria: Public health facilities that have outpatient pharmacies or dispensaries and private sector licensed retail pharmacies (closer to public health facilities) that are expected to stock psychotropic medicines were included in the study

Exclusion Criteria: Public Health facilities that only stock a small numbe[illegible] emergency psychotropic medicine; and pharmacies in private clinics and hospitals or health facilities operated by private companies, such as mining companies, were excluded. Furthermore, [illegible]g stores were excluded from the study.

Sampling Strategy

The residents of Addis Ababa are economically heterogeneous. During this study, the city was administratively divided into 10 Sub-Cities. As shown in Table 3, the ten sub-cities are stratified into three subgroups based on their relative per capita income and poverty status (32). Accordingly, the economic variations of the population have been addressed by selecting one representative sub-city from each sub-group using a simple random sampling.

Table 1: Grouping of sub-cities in Addis Ababa based on income status and poverty

Burden of poverty	Sub Cities	Per capita income in birr/year	Rank
Group 1: 1%-10%	Bole	15551	1
	AkakiKality	13448	2
	Yeka	12146	4
Group 2: 11%-20%	Kirkos	12265	3
	Gullele	11008	6
	Nefas Silk Lafto	10264	7
	KolfeKeranyo	11059	5
Group 3: >20%	Lideta	8448	8
	Arada	8101	9
	Addis Ketema	7226	10

Source: (32).

In addition to economic status, accessibility to essential psychotropic medicines has also been affected by the type of health sector. The two main types of health sectors in Addis Ababa are private and public. Thirty retail outlets for each sector were taken as an optimal sample size as per WHO's recommendatio[illegible] Stratified random sampling was used to select sub-cities. In each sub-city retail outlets were visited until the allocated number was reached. Besides, the private outlets were selected based on their proximity to primary, secondary, and tertiary public health facilities surveyed (Table 2)

Table 2: Distribution of health facilities in each sub-city of Addis Ababa, 2019.

Subgroups	All Sub Cities	Study Population		Selected Sub cities	Sample Size		Total
		Public	Private		Public sector	Private sector	
Group:1	Bole	10	65	Bole Sub-city	10	14	24
	AkakiKality	10	38				
	Yeka	15	71				
Group:2	Kirkos	10	30	Kolfe Sub city	10	8	18
	Gullele	13	130				
	Nefas Silk	10	26				
	KolfeKeranyo	11	38				
Group:3	Lideta	8	20	Addis Ketema	10	8	18
	Arada	13	102				
	Addis Ketema	10	38				
				**Total**	**30**	**30**	**60**

Sources: Addis Ababa Health Bureau's FMHACA 2019.

Data Collectors, Procedures, and Collection Tool

Two trained pharmacists were employed for this study as data collectors. Data on the price and availability of psychotropic medicines was collected by physically inspecting the stock of the originator brand (OB) medicines and their lowest price generic equivalents (LPG) on the day of the survey using a standardized WHO data collection form (29). For each medicine listed, information regarding the manufacturer, the pack size, and the pack price was recorded for both products. The procurement price was gathered from public-sector procurement agency. The prices were converted to US dollars using the buying exchange rate on 30 July/2019, the first day of data collection, i.e., 1USD = 29.0256 ETB.

Data Quality Assurance

The medicine's price data collection form was pre-tested in 20% of the total sample size (n=60) before the actual data collection. The medicines' list, strength, and dosage forms were reviewed following the pre-test. Also, during the actual data collection, the questionnaire was revised for completeness of the forms and accuracy of unit prices each day. Double data entry and workbook auto checker were also employed to identify any discrepancies and to ensure the accuracy of the data entry process. Descriptive statistics were also used to clean the raw data.

Data Analysis

WHO/HAI price workbook-part-I and the Statistical Package for the Social Sciences (SPSS) version 25 were used to enter, edit, analyze and summarize the data. In order to measure the outcomes of this study, the following definitions were used.

In this study, medicine availability was defined as the presence of the survey medicines at the specified strength and dosage form in selected retail outlets on the day of the survey. Availability was determined as the mean availability of individual medicines, group of medicines, product types (originator brand vs. generic), of medicines between sectors. The following ranges were used to describe percentage availability (33).

- < 30% = very low
- 30–49% = low
- 50–80% = fairly high
- >80% = high

In the analysis of price data, median prices of medicines in local currency were used. MPRs could not be calculated until at least four retail patient prices were available for the medicine in question from each sector. The international price guide indicator of Management Sciences for Health (MSH) has been used for price comparisons (34). Where no supplier prices were available, median buyer unit price was used.

The median price ratio was obtained by dividing the local medicine price by the international reference price. Thus, the ratio tells us how much greater or less the local medicine price is to the international reference price. However, there are no universally accepted interpreting MPRs due to the difference in medicine price components and the different market systems in various countries. For this study, the following MPRs cut-off points were used to indicate acceptable local price ratios based on the definition used in similar surveys performed in Ethiopia and elsewhere with similar methodology (35, 36).

- Public sector procurement price: MPR ≤ 1
- Public sector patient price: MPR ≤ 1.5
- Private retail pharmacy patient price: MPR ≤ 2

The affordability of standard treatments for five different mental health conditions was analyzed. The total monthly dose was determined by multiplying each medicine's commonly prescribed daily dose by 30 days. The affordability of treatments was assessed by considering the recently updated net salary of the lowest-paid government worker from the Federal Civil Service Authorities of Ethiopia, which came into effect on 8 July 2019, which was 973 birr/month, or 32.28$/month. Accordingly, the daily wage was determined by dividing the net salary for 30 days, ETB 32.43/day, or USD 1.12/day. The treatment that cost only 1-day income or less was deemed affordable (29).

Results

Availability of Psychotropic Medicines

The mean availability for 20 LPG psychotropic medicines in Addis Ababa city was found to be 24.3%. Out of the 20 psychotropic medicines surveyed, only the originator brand of carbamazepine was found across the 60 outlets, and its mean availability was 2.4%.

The overall mean availability of the basket of medicines across the sample of 30 public medicine outlets was 28.7% for the LPG and 2.8% for the OB. Among the 20 essential psychotropic medicines surveyed, one OB and 17 LPG medicines were found across the sampled public sector medicine retail outlets.

Medicines like alprazolam 0.5 mg tab, clozapine 25 mg tab, and lamotrigine 50 mg tablet were not found throughout the sampled public medicine retail outlets, and the availabilities for most of the surveyed medicines were below 50%. The percentage availability for the OB of carbamazepine was 56.70% which was much greater than the availability of its LPG (3.3%).

Similarly, the finding showed that neither the LPG nor the OBs of 11 medicines was found in any private medicine retail outlets in the private sector. However, the remaining nine medicines were found with availability ranging from 3.3% to 90.0% in medicine retail outlets in this sector. The overall mean availability of these psychotropic medicines in the private sector was 19.8% for the LPG and 2.0% for the OB medicines. The availability of Tegretol was 40%, but its LPG was 20%. Besides this, it was only fluoxetine and sodium valproate out of the 20 LPG medicines with adequate availability, > 80%, as shown in Table 3.

Table 3: Availability of LPG medicines in public and private retail outlets (n=60), Addis Ababa, 2019.

Medicines name, strength and dosage form	Public retail outlets (n=30)	Private retail outlets (n=30)
Alprazolam 0.5 mg tab	0.0	0.0
Amitriptyline 25 mg tab	90.00%	56.70%
Carbamazepine 200 mg tab	3.30%	20.00%
Chlorpromazine 100 mg tab	76.70%	13.30%
Clomipramine 25 mg cap	3.30%	0.0
Clozapine 25 mg tab	0.0	0.0
Diazepam 5 mg tab	80.00%	0.0
Fluoxetine 20 mg cap	56.70%	83.30%
Fluphenazine 25 mg/ml inj	13.30%	0.0
Haloperidol 2 mg tab	46.70%	0.0
Imipramine 25 mg tab	33.30%	0.0
Lamotrigine 50 mg tab	0.0	50.00%
Lithium CO3 300 mg cap	6.70%	0.0
Olanzapine 5 mg tab	20.00%	3.30%
Phenobarbital 30 mg tab	73.30%	33.30%
Phenytoin 100 mg tab	26.70%	0.0
Risperidone 1 mg tab	16.70%	46.70%
Sertraline 50 mg tab	13.30%	0.0
Sodium Valproate 200 mg tab	10.00%	90.00%
Trifluoperazine 5 mg tab	3.30%	0.0

As per the level of healthcare facilities sampled in the public sector, essential psychotropic medicines' availability was also analyzed. The mean availability of the LPG medicines in the health centers and hospitals were 24.2% and 51.0%, respectively. Similarly, the mean availability of the OB was 2.4% and 5% for health centers and hospitals, respectively. Besides, about 85% of the surveyed LPG medicines

were observed only in one specialized mental hospital, but the mean availability of the LPG in the 29 non-mental healthcare facilities was 26.7%. Despite the number of public health facilities found at various healthcare levels, the mean availability of OB medicines was meager. Whereas the availability of LPG medicines was improved as the level of care has increased.

As shown in Figure 1, the availability of at least one essential psychotropic medicine from each therapeutic class was only observed only in six public medicine retail outlets, and no retail outlet was found in the private sector that stock at least one psychotropic medicine from each of the five therapeutic classes. The majority of the retail outlets in both sectors (n=28/60) were stocking at least one medicine from the three classes alone.

Figure 1: Availability of at least one medicine from each therapeutic class per retail outlet, Addis Ababa, 2019

Price of Psychotropic Medicines

Public Procurement Prices

The procurement prices of one originator brand and 16 lowest price generic medicines were obtained. About half of the LPG medicines (n=8/16) were being procured $\leq$ 1 MPR; whereas the other LPG were procured > 1 MPRs. The MPRs for OB and LPG of carbamazepine 200mg tab were 4.36 and 1.68 times the international reference price, respectively. Also, as shown in Table 4, the procurement price data of 4 LPG medicines were not found during the survey. The summary findings of the procurement prices for 16 LPG showed that the minimum MPR was 0.25, the maximum MPR was 4.83, and the median MPR was 0.96.

Table 4: Public Sector Procurement Prices of medicines, Addis Ababa, 2019

No.	Medicine Name	Medicine Type	Median Price Ratio	Median Price
1	Alprazolam 0.5 mg tab	LPG	1.78	0.83
2	Amitriptyline 25 mg tab	LPG	1.94	0.47
**3**	Carbamazepine 200 mg tab	**OB**	4.36	2.34
3	Carbamazepine 200 mg tab	LPG	1.68	0.90
4	Chlorpromazine 100 mg tab	LPG		
5	Clomipramine 25 mg cap	LPG	0.39	0.54
6	Clozapine 25 mg tab	LPG		
7	Diazepam 5 mg tab	LPG	0.25	0.07
8	Fluoxetine 20 mg cap	LPG	2.66	0.80
9	Fluphenazine 25 mg/ml inj	LPG	0.65	15.77
10	Haloperidol 2 mg tab	LPG		
11	Imipramine 25 mg tab	LPG	0.73	0.38
12	Lamotrigine 50 mg tab	LPG	4.83	2.69
13	Lithium CO_3 300 mg cap	LPG	1.03	0.85
14	Olanzapine 5 mg tab	LPG	0.26	0.72
15	Phenobarbital 30 mg tab	LPG	0.45	0.10
16	Phenytoin 100 mg tab	LPG	1.54	0.47
17	Risperidone 1 mg tab	LPG	3.08	3.35
18	Sertraline 50 mg tab	LPG	0.89	0.60
19	Sodium Valproate 200 mg tab	LPG	0.60	1.22
20	Trifluoperazine 5 mg tab	LPG		

**Statistics**	**LPG**	**OB**
Number of medicines included	16	1

Median MPR	0.96	4.36
25 %ile MPR	0.44	4.36
75 %ile MPR	1.7	4.36
Minimum MPR	0.25	4.36
Maximum MPR	4.83	4.36

Retail Prices of Medicines in Public and Private Sectors

The patient prices of the surveyed psychotropic medicines in both sectors are depicted in Table 5 below. MPR prices for 8 LPG medicines in public and 12 LPG medicines in private retail outlets were not determined. This is because either these medicines were found in less than four outlets, or else these medicines might not be found at all in any sampled outlets of both sectors.

In the public sector, the summary findings in table 5 below showed that the median MPR amongst the 12 LPG medicines included in the price data analysis was 1.53. In contrast to the LPG, the MPR price for the OB of carbamazepine was 5.21, the same value throughout the remaining summary statistics since this patient price data was computed for one OB product only. In the private sector, 8 LPG medicines were included to analyze patient prices, and the median MPR amongst the 8 LPG medicines was 5.89. The median MPR price for the OB of carbamazepine was 11.17. Like the public sector, only the OB of the carbamazepine was also found in the private sector.

Table **5**: Prices of medicines in public and private retail outlets, Addis Ababa, 2019

No.	Medicine Name	Medicine Type	Public (n=30)		Private (n=30)	
			**MORE**	**Median Price**	**MORE**	**Median Price**
1	Amitriptyline 25 mg tab	LPG	2.54	0.62	9.02	2.20
2	Carbamazepine 200 mg tab	LPG			4.89	2.63
3	Carbamazepine 200 mg tab	**OB**	5.21	2.8	11.17	6.00
4	Chlorpromazine 100 mg tab	LPG	0.6	0.25	1.08	0.45
5	Diazepam 5 mg tab	LPG	0.64	0.18		
6	Fluoxetine 20 mg cap	LPG	3.51	1.05	10.37	3.10
7	Fluphenazine 25 mg/ml inj	LPG	1.43	34.73		
8	Haloperidol 2 mg tab	LPG	1.64	1.04		
9	Imipramine 25 mg tab	LPG	1.33	0.7		
10	Lamotrigine 50 mg tab	LPG			24.28	13.53
11	Olanzapine 5 mg tab	LPG	0.52	1.42		
12	Phenobarbital 30 mg tab	LPG	0.92	0.2	6.89	1.50
13	Phenytoin 100 mg tab	LPG	2.74	0.83		
14	Risperidone 1 mg tab	LPG	6.43	7	4.64	5.05
15	Sertraline 50 mg tab	LPG	1.63	1.11		
16	Sodium Valproate 200mg tab	LPG			3.97	8.00

**Summary statistics**	**Public Sector**		**Private Sector**	
Type of Medicines	LPG	OB	LPG	OB
Number of medicines included	12	1	8	1
Median MPR	1.53	5.21	5.89	11.17
25 %ile MPR	0.85	5.21	4.47	11.17
75 %ile MPR	2.59	5.21	9.36	11.17
Minimum MPR	0.52	5.21	1.08	11.17
Maximum MPR	6.43	5.21	24.28	11.17

Affordability of Psychotropic Medicines

As table 6 below shows, 5 LPG were costing ≤ 1 day's wage for treating the specified mental disorders in the public sector. However, the remaining LPG required more than one day's wage to buy the standard treatments in the public health facilities. For treating epilepsy, the OB of carbamazepine was not affordable in both the public and private medicine retail sectors. Similarly, some LPG treatment costs were surprisingly high when purchased in the private sector. For instance, treating epilepsy with lamotrigine 50 mg tablets required 25 days' wages, while treating bipolar disorders with sodium valproate costs 22.2 days' wages in the private retail pharmacies. In the private sector, only the cost of chlorpromazine was affordable.

Table 6: Affordability of Standard Treatments, Addis Ababa, 2019.

Mental Disorders	Standard Treatments				Public Sector		Private Sector	
	Medicine, its Strength and Dosage Form	[illegible]eatment Duration (in Days)	Total doses per treatment	Product Type	Median Treatment Price	Days' Wages	Median Treatment Price	Days' Wages
Depression	Amitriptyline 25 mg tab	30	90	LPG	55.80	1.7	198.00	6.1
	Fluoxetine 20 mg cap	30	30	LPG	31.50	1.0	93.00	2.9
	Imipramine 25 mg tab	30	60	LPG	42.00	1.3		
	Sertraline 50 mg tab	30	30	LPG	33.18	1.0		
Epilepsy	Carbamazepine 200 mg tab	30	90	Brand	252.00	7.8	540.00	16.7
				LPG			236.25	7.3
	Lamotrigine 50 mg tab	30	60	LPG			811.80	25.0
	Phenobarbitone 30 mg tab	30	90	LPG	18.00	0.6	135.00	4.2
	Phenytoin 100 mg tab	30	90	LPG	74.43	2.3		
Psychosis	Chlorpromazine 100 mg tab	30	60	LPG	15.00	0.5	27.00	0.8
	Fluphenazine Inj 25 mg/ml	30	1	LPG	34.73	1.1		
	Haloperidol 2 mg tab	30	60	LPG	62.40	1.9		
	Olanzapine 5 mg tab	30	90	LPG	127.58	3.9		
	Risperidone 1 mg tab	30	30	LPG	210.00	6.5	151.50	4.7
Anxiety	Diazepam 5 mg tab	10	10	LPG	1.78	0.1		
Bipolar	Sodium valproate 200 mg tab	30	90	LPG			720.00	22.2

Discussion

In the subsequent sections, the findings of this study in relation to the medicines' availability, prices, and affordability have been discussed.

This study found that the overall mean availability of the LPG psychotropic medicines in Addis Ababa was 24.3%. This value of mean percentage availability is below 30%, indicating a very low availability of essential psychotropic medication for the study area population (33). Similarly, a national pharmaceutical sector assessment in 2016 indicated a very low availability of medicines for non-communicable diseases, including mental disorders (36). Furthermore, this figure was far from the optimum availability indexes recommended by WHO which is above 80% (37). This might be due to a lack of attention to mental health problems and the availability of limited resources for improving access to essential psychotropic medicines (37, 38, 39).

In the public sector, the mean availability of the LPG psychotropic medicines was 28.7%. This was generally very low but higher in hospitals (51%) than in health centers (24.2%). Comparing this result to the previous study, the median availability of medicines for chronic illnesses, including mental disorders in the hospitals (81.8%), was more than two times the median availability in the health centers (36.4%). In comparison, the median availability for other medicine groups, for instance, anti-infective medicines, was 87.5% in both the health centers and hospitals (36). This might imply an inequity of access to essential medicines for mental disorders at all healthcare levels. When comparing the percentage availability of individual medicines, lower availability was observed in this study than in the previous survey (38). This might imply no improvement in the supply side of access to essential psychotropic medicines while increasing demands for more mental health medicines were evident (15).

Similarly, in the private sector, the mean availability of LPG psychotropic medicines was 19.8%, lower than what was found in the public sector covered in this study. This was also similar to the finding of a previous national study conducted in Ethiopia (36). However, a study conducted in the private sector of Malawi, which included selected psychotropic medicines, reported that the availability of these medicines

was higher than the private retail outlets of this study. For instance, the availability of diazepam in the private sector in Malawi was 100% (40). However, in the present study, diazepam was not found in all private facilities of Addis Ababa. The highly controlled nature of the medications and the fact that these medications are less prescribed in the private sector might have contributed to their lower availability.

Furthermore, the mean availability in the private sector in Addis Ababa was less than the finding in the private sector of Saud Arabia (41). This variation might have resulted from this study's dose and strength-specific feature. The overall mean availability in both sectors was far from the recommended minimum availability cut-off point (29).

Concerning availability from each therapeutic class, only 20% of the public retail outlets (n=6/30) had at least one medicine from the five therapeutic groups of psychotropic medicines. Of these public retail outlets that stock at least one medicine from each class, most were hospitals (n=5/6). Most retail outlets were stocking at least one essential psychotropic medicine from the three classes alone from both sectors. This result was lower than the result found in the public sector of Mozambique, where 45.8% of the health facilities had at least one medicine from each class (26). This implies that the minimum availability of at least one medicine from each therapeutic class, as suggested by WHO, was not achieved yet (42). This limit is essential for providing minimum care to people with a mental health condition (43). Besides, about 85% of the surveyed LPG medicines were available only in one specialized mental hospital. Similarly, a WHO study and a study done in Mozambique indicated that psychotropic medicines from each therapeutic category were more readily available in the mental health facilities than in non-specialized healthcare facilities (26.7%) (17, 26), indicating the non-decentralization of the mental healthcare service to all levels of the healthcare in country's health systems.

Regarding procurement prices, out of the 20 LPG medicines surveyed, the prices of 16 LPG medicines were found in the Agency. Based on this procurement price data, the median MPR for the 16 LPG was 0.96 MPR, which was within the range of acceptable public procurement prices. However, when looking

at the MPRs of each medicine, there were medicines purchased at ≥1MPR. While eight medicines were procured below 1 MPR (ranging from 0.25-0.89), the other halves were procured above 1 MPR (ranging from 1.03-4.83) compared with the IRPs (34). This data suggests the public procurement agency was showing an efficient procurement in 50% of the LPG medicines for which prices were obtained. When comparing the procurement prices of selected medicines (carbamazepine and phenytoin) with the previous study, the MPRs of carbamazepine and phenytoin increased by 184.75% 258.14% respectively from the MPRs of carbamazepine=0.59 and phenytoin=0.43 in 2004 (38). For some of the medications indicated, the purchasing efficiency of the public procurement sector in 2004 was better than in 2019. The need to explore the underlying factors that cause such expensive medicine procurement prices is imperative. Maintaining the public procurement prices within the acceptable range (≤1MPR) is very decisive, particularly for low-income countries like Ethiopia, in improving access to key medicines for mental disorders (33).

Concerning patient prices, the median patient price for 12 LPG out of the 20 surveyed psychotropic medicines in the public sector retail outlets was found to be 1.53 MPR. This price was slightly above the acceptable MPR price cut-off point used in this study (≤1.5MPR), but it was generally showing an improvement in the public sector retail outlets. However, for 6 LPG, the public patient prices were clearly above 1.5MPR (ranging from 1.63-6.43 MPRs). These high prices are resulted from the same medicines purchased with costs above 1MPR during the procurement, except sertraline 50mg tablet that was expensive due to the excessive mark-up (83.15%). This data substantiated the fact that procurement prices directly affect the patient prices of medicines in the public health facilities and the mark-ups that significantly affect the patient price (23). The median MPR price of the OB for carbamazepine in the public sector was 5.21 MPR. However, the mark-up difference between the procurement price and patient price of the OB was 19.7% which is below the average mark-up of the study area (22.04%) (36). This might indicate that the government seems to apply a regressive mark-up pricing strategy (using lower

mark-up for higher-priced products rather than fixed percentage mark-ups) in the public retail outlets (44).

Alternatively, the median MPR of patient prices in the private sector across 8 LPG medicines was 5.89 MPR which was more than the acceptable cut-off price used in this study and higher than four MPR of the IRPs (29). Then, this median MPR price showed 284.97% higher prices in the private sector than the median MPR of the patient price in the public retail outlets (1.53 MPR). Again, the MPR prices for 7 LPG out of 8 LPG (87.5%) ranged from 3.97 MPR - 24.28 MPR, which were > 2 MPR. So, it was only the MPR price of chlorpromazine 100 mg tablet (1.08 MPR) which was found within the acceptable price range for private retail outlets (≤ 2 MPR). Consequently, the low availability of medicines at public medicine retail outlets could directly impact access to essential psychotropic medicines as patients are then forced to buy these medicines from private pharmacies. The better availability in the public sector would pressure the private sector to lower the price of these generic medicines due to competition (45). In addition, the higher mark-up prices in the private sector might be due to the lack of medicine price regulating mechanisms (46) and the variation in the type of procurement methods used between the two sectors (47, 48). Similarly, the MPRs for amitriptyline and fluoxetine were increased by 215.38% and 625.17%, respectively, compared to the previous study in the private sector (36).

When looking at the affordability, most of the psychotropic medicines found in both sectors were less affordable. However, 5 of 12 LPG medicines were found to be affordable in the public retail outlets meeting the affordability targets of WHO (29). The other 7 LPG were unaffordable, requiring 1.1 to 6.5 days' wages to cover a month of treatment. This shows that more than 58% of LPG found in the public outlets were unaffordable for the lowest-paid unskilled government workers in Addis Ababa. So patients who could not afford such out-of-pocket costs would forgo the treatment that leads to increasing the burden of mental illness (49, 15). Higher medicines' prices and the low income of the lowest-paid workers contributed for the unaffordability of medicines for mental illnesses. The need to employ one of

the feasible price-reducing interventions on generic medicines in the public sector retails has been considered the only way to improve access for many low-income patients (44).

In the private outlets, only chlorpromazine 100 mg tablet was affordable out of the 8 LPG medicines found in this sector. The present study showed that 87.5% of the existing treatments for mental disorders in private retails were non-affordable to many people with mental health conditions in Addis Ababa City (32). Equally, the cost of medicines in private retail outlets documented in this study required more days' wages than previous studies done in different countries (38, 50). The prices of key medicines, including essential psychotropic ones, should not be left solely to market forces (10). Otherwise, in a population with low-income levels and high medicine prices, it would mean that access to medicines is only affordable to wealthy segment of the population (45).

Moreover, costs of the OB were unaffordable in both sectors. In this regard, 16.7 days wage and 7.8 days wage is required to buy 30 days' supply from private and public retail outlets, respectively. This data was similar to a study done by WHO expert groups (51). However, as the affordability data in this study was estimated from one OB alone, this report might be less conclusive about the affordability of OBs for psychotropic medicines. Moreover, even treatments that seem affordable in this report are too costly, at least for 18% of the population in Addis Ababa who are earning less than the reference group (32).

Finally, this study provides an essential picture about access to psychotropic medications. Stakeholders were involved during the proposal development, and their suggestions were included in the actual study. Additionally, the clinical importance of the surveyed medicines has also been triangulated between the national essential list, the national standard treatment guideline, MSH 2015 price lists, and WHO essential lists. Moreover, variations on the type of health sectors and the economic status of the general population for access to medicines in the study area were considered.

However, this study has not been without limitations. The data on the availability of medicines was collected at a specific point in time. Thus, it does not reflect the average monthly or yearly availability of essential psychotropic medicines at the individual pharmacy outlets. Besides, some psychotropic medicines studied, such as carbamazepine, phenobarbitone, phenytoin, risperidone, and sodium valproate, were found in different strengths and formulation types other than what was specified in the medicine price data collection form. As a result, such medicines available in different dosage forms and strengths were excluded from this study. Therefore, these medicines' non-availability and lower availability may not give sense because they are available but with different strengths and dosage forms. Lastly, when other costs were also considered, treatments that appear relatively affordable in this study might represent an overestimation of affordability.

Conclusion

The mean availability of the LPG psychotropic medicines was generally very low in Addis Ababa–yet it was better in the public health facilities than in private retails. Availability was lower in health centers than in hospitals. Most retail outlets were stocking at least one medicine from three classes alone in both sectors. Only the OB of carbamazepine was found in all medicine outlets with extremely low availability.

The public procurement prices for half of the surveyed psychotropic medicines were high. Moreover, in both sectors, the patient prices for more medicines were high, and the costs for most of the standard mental treatments were considered unaffordable not only to the lowest-paid government workers but also to most populations in Addis Ababa living under the poverty line, especially for the private outlets.

List of Abbreviations

FMHACA: Food, Medicine and Healthcare Administration and Control Authority; HIA: Health Action International; IOM: Institute of Medicine; MOH: Ministry of Health; MSH: Management Sciences for

Health; OECD: Organization for Economic Cooperation and Development; PFSA: Pharmaceutical Fund and Supply Agency; UN: United Nations; WHO: World Health Organization.

Declarations

Ethical approval and consent to participate: The study was conducted after the ethical approvals obtained from the Ethics Review Board of the School of Pharmacy, Addis Ababa University and Addis Ababa City Administration Health Bureau. Besides, each participant provided informed verbal consent to participate in the study.

Consent for publication: N/A

Availability of data and materials: The data sets generated and/or analyzed during the current study are not publicly available due ethical restrictions but are available from the corresponding author on reasonable request.

Competing interests: All authors declared that there are no conflicts of interest.

Funding: None

Authors' contributions: MTA designed the study, supervised the data collection, did the analysis and drafted the manuscript; TGF conceived the study and both TGF and MA involved in the design and critical review of the manuscript. All authors read and approved the final manuscript

Acknowledgments

The authors would like to acknowledge the heads of all institutions that provided permission and study participants for giving information and having made relevant documents available for review.

References

1. WHO 2001a. Mental health: new understanding, new hope. Geneva: World Health Organization, 2001.
2. WHO 2005a. Promoting mental health: concepts, emerging evidence, practice. A report of the World Health Organization, in collaboration with the Victorian Health Promotion Foundation and the University of Melbourne Geneva: World Health Organization, 2005.
3. WHO. Mental health action plan 2013-2020. Geneva: World Health Organization, 2013.
4. MOH/WHO. mhGAP in Ethiopia: Proof of Concept. Addis Ababa: Ministry of Health, 2013.
5. MOH. National Mental Health Strategy 2012/13 - 2015/16. Addis Ababa: Ministry of Health, 2013.
6. Fekadu, A., Hanlon, C., Gebre-Eyesus, E., Agedew, M., Solomon, H., Teferra, S., et al. The burden of mental disorders and unmet needs among street homeless people in Addis Ababa, Ethiopia. *BMC Medicine 2015;* **12**(138).
7. Alem, A., Kebede, D., Fekadu, A., Shibre, T., Fekadu, D., Beyero, T., et al. Clinical course & outcome of schizophrenia in a predominantly treatment-naive cohort in rural Ethiopia. Schizophr Bull. 2009; 35(3): 646-654.
8. WHO 2003a. Investing in mental health. Department of mental health and substance dependence. Geneva: World Health Organization, 2003.
9. Sathiyasusuman, A. Mental Health Services in Ethiopia: an emerging public health issue. Public Health 2011; 125(10):714-6.
10. WHO 2005b. Improving access & use of psychotropic medicines. Mental Health Policy and Service Guidance Package. Geneva: World Health Organization, 2005.
11. WHO 2009a. Pharmacological Treatment of Mental Disorders in Primary Health Care. Geneva: World Health Organization, 2009.
12. Balasubramaniam, K. Access to Essential Medicines as a Human Right. The Politics of Medicines, 2012.
13. WHO 2017a. Advancing the right to health: the vital role of law. Geneva: World Health Organization, 2017.
14. Mccrone, P., Dhanasiri, S., Patel, A., Knapp, M. & Lawton-Smith. Paying the price: The cost of mental health care in England to 2026. London: King’s Fund, 2008.

15. Johansson, KA., Strand, KB. & Fekadu, A. Health Gains and Financial Protection Provided by the Ethiopian Mental Health Strategy: an Extended Cost-Effectiveness Analysis. Health Policy and Planning 2016; **32**(3): 376-383.
16. WHO 2001b. Atlas: Mental health resources in the world. Geneva: World Health Organization, 2001.
17. WHO. Mental health systems in the Eastern Mediterranean Region report based on the WHO assessment instrument for mental health systems. EMRO Technical Publications Series. Geneva: World Health Organization, 2010.
18. OECD. Focus on health: making mental health count. Paris: OECD Publishing, 2014.
19. IOM. Improving access to essential medicines for mental, neurological, and substance use disorders in sub-Saharan Africa: workshop summary, Washington, DC: The National Academies Press, 2014.
20. Solomon, A., Mihretie, G. & Tesfaw, G. 2019. The prevalence & correlates of common mental disorders among prisoners in Addis Ababa: An institution-based cross‑sectional study. BMC Research Notes 2019; 12(1): 394.
21. Rathod, S., Pinninti, N., Irfan, M., Gorczynski, P., Rathod, P., Gega, L. et.al. Mental health service provision in low- and middle-income countries. Health Services Insights 2017; 10:1-7.
22. WHO 2017b. Improving access to & appropriate use of medicines for mental disorders. Geneva: World Health Organization, 2017.
23. WHO. The World medicines situation: Medicines prices, availability & affordability. 3rd Edition. Geneva: World Health Organization, 2011.
24. Alem., A, Desta, M. & Araya, M. Mental Health in Ethiopia. *Ethiopian Journal of Health Development* 1995; **9**(1):47-62.
25. Ayano, G. Primary Mental Health Care Services in Ethiopia: Experiences, Opportunities, and Challenges from East African Country. *Neuropsychopharmacology & Mental Health 2016*; 1(4).
26. Bradley, H., Wagenaar, Stergachis, A., Rao, D., Hoek, R., Cumbe, V., Napu´, M. et al. The availability of essential medicines for mental healthcare in Sofala, Mozambique. *Glob Health Action 2015;* **8**.
27. Eaton, J. Ensuring access to psychotropic medication in sub-Saharan Africa. *Afr J Psychiatry 2008;* **11**(3): 179-181.
28. Central Statistics Agency. Population projections for Ethiopia 2007-2037. Addis Ababa: Central Statistics Agency, 2013.

29. WHO & HAI. Measuring medicine prices, availability, affordability, and price components. Second edition. Geneva: World Health Organization & Health Action International, 2008.
30. FMHACA. National essential medicine lists. Fifth Edition. Addis Ababa: Food Medicine & Healthcare Administration & Control Authority, 2015.
31. WHO. World Health Organization Model List of Essential Medicines. 21th Edition. Geneva: World Health Organization, 2019.
32. Gebre-Egziabher, T., Woldehanna, T., Gurmu, E. & Amdework, E. Poverty level assessment of Addis Ababa City. Addis Ababa: Institute of Development & Policy Research (IDPR) Addis Ababa University, 2015.
33. Gelders, S., Ewen, M., Noguchi, N. & Laing, R. Price, availability, and affordability: an international comparison of chronic disease medicines. A background report prepared for the WHO Planning Meeting on the Global Initiative for Treatment of Chronic Diseases held in Cairo in December 2005 Cairo: World Health Organization & Health Action International, 2006.
34. MSH. International Medical Products Price Guide, 2015 Edition. Medford, Mass: Management Sciences for Health, 2016.
35. Wang, X., Fang, Y., Yang, S., Jiang, M., Yan, K., Wu, L. et al. Access to pediatric essential medicines: A survey of prices, availability, affordability & price components in Shaanxi Province, China. PLoS ONE 2014; 9:1-7.
36. FMHACA, PFSA & WHO. Pharmaceutical Sector assessment in Ethiopia. Addis Ababa, 2017.
37. WHO 2009b. Mental health systems in selected LMICs: a WHO-AIMS cross-national analysis. Geneva: World Health Organization, 2009.
38. Cameron, A., Bansal, A., Dua, T., Hill, S. R., Moshe, S. L., Mantel-Teeuwisse, AK. et al. Mapping the availability, price, and affordability of antiepileptic drugs in 46 countries. *Epilepsia 2012;* **53**(6):962-969.
39. WHO. Mental Health 2017 Atlas. Geneva: World Health Organization, 2018.
40. Khuluza, F. & Haefele-Abah, C. The availability, prices, and affordability of essential medicines in Malawi: A cross-sectional study. PLoS ONE 2019; 14(2).
41. Al-Ruthia, YS., Mansy, W., Barasin, M., Ghawaa, YM., Alsultan, M., Alsenaidy, MA. et al. Shortage of psychotropic medications in community pharmacies in Saudi Arabia: Causes and solutions. Saudi Pharm J *2017;* **25**:744-749.
42. WHO 2005c. World Health Organization assessment instrument for mental health systems. WHO-AIMS Version 2.2. Geneva: World Health Organization, 2005.

43. Huskamp, HA. Prices, profits, and innovation: Examining Criticisms of New Psychotropic Drugs' Value. Health Affairs 2006; **25**(3).
44. WHO. Guideline on country pharmaceutical pricing policies. Geneva: World Health Organization, 2015.
45. UN. The role of competition in the pharmaceutical sector and its benefits for consumers. Geneva: UNCTAD secretariat. TD/RBP/CONF.8/3, 2015.
46. Ministry of Health. Ethiopian National Drug Policy. National drug policy of the transitional government of Ethiopia. Addis Ababa: Ministry of Health. 1993.
47. WHO. Operational principles for good pharmaceutical procurement. Geneva: World Health Organization, 1999.
48. Ashigbie, PG., Azameti, D. & Wirtz, VJ. Challenges of medicines management in the public and private sector under Ghana's National Health Insurance Scheme – A qualitative study. Pharmaceutical Policy and Practice *2016;* **9**: 6**.**
49. Rowan, K., Mcalpine, DD. & Blewett, LA. Access & cost barriers to mental health care, by insurance status, 1999–2010. Health Affairs 2013; 32.
50. Saeed, A., Saeed, H., Saleem, Z., Fang, Y., Zaheer-Ud- & Babar, D. Evaluation of prices, availability and affordability of essential medicines in Lahore Division, Pakistan: A cross-sectional survey using WHO/HAI methodology. PLoS ONE 2019; 14(4).
51. Ewen, M., Zweekhorst, M., Regeer, B. & Laing, R. Baseline assessment of WHO's target for both availability & affordability of essential medicines to treat non-communicable diseases. *PLoS ONE 2017;* **12**(2).

Figure Click here to access/download;Figure;Figures.docx

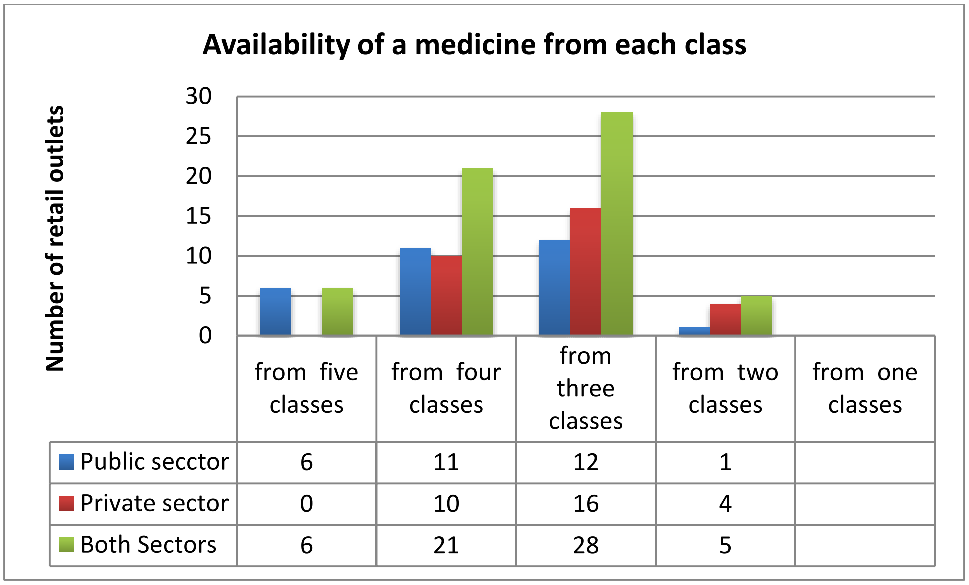

Figure 1: Availability of at least one medicine from each therapeutic class per retail outlet, Addis Ababa, 2019
