# Supplementary material for: Access to essential psychotropic medicines in Addis Ababa: A cross-sectional study
Source: PLoS One. 2023 Jul 14;18(7):e0283348. doi: 10.1371/journal.pone.0283348 (PMC10348529; doi:10.1371/journal.pone.0283348)
Supplement: Attachment — Submitted filename: Review comments - Access to Essential Psychotropic Medicines in Addis Ababa.docx [file pone.0283348.s004.docx]

**Access to Essential Psychotropic Medicines in Addis Ababa: A Cross-Sectional Study**

This article is recommended for publication after improvement of the methods and results presentation sections. The study is scientifically relevant, the methodology employed is appropriate but not clearly described, and the way results are presented needs to be improved.

A. Major comments
I. General comments:
   1. Carefully read the “Instructions for authors” concerning the format and how to cite references in the text. Ideally, use an automatic referencing system (e.g. Mendeley) and select the journal’s style.
II. References:
    1. for all non-journal article references, provide the URL link and the date you lastly accessed it, to help reviewers check the correctness of the information.
III. Methods:
     1. “1919 health facilities in Addis Ababa; of which 110 of them were public healthcare facilities and 558 were private retail pharmacies.” How about the other 1251 health facilities?
     2. “The study population was 668 medicines”. How did you move from 1919 to 668 health facilities?
     3. “The sample size was determined based on the WHO/HAI manual (29). Accordingly, 60 retail outlets; 30 each from the public and private sectors, were included.” Please, describe your methods clearly to help the readers understand what and how you have done. How did you come up with 60 from 1919 health facilities?
     4. “Inclusion Criteria: Public health facilities that have outpatient pharmacies or dispensaries and private sector licensed retail pharmacies (closer to public health facilities) that are expected to stock psychotropic medicines were included in the study.
        Exclusion Criteria: Public Health facilities that only stock a small number of emergency psychotropic medicine; and pharmacies in private clinics and hospitals or health facilities operated by private companies, such as mining companies, were excluded. Furthermore, drug stores were excluded from the study.” Please, consult your teachers of research methodology to understand the meaning of exclusion criteria: you can’t take beans A and B from a basket, while beans A and B have not been in the basket! Correctly write this section.
     5. “Thirty retail outlets for each sector were taken as an optimal sample size as per WHO’s recommendation.” Please, provide a reference with the URL link for this statement!
     6. Table 2: why did you sample more health facilities from Bole sub-city than from Kolfe and Addis Ketema (24/60)?
B. Minor comments
I. Entire manuscript:
   1. The text lines should be numbered to ease the review with reference to the line numbers.
II. Title page:
    1. Author 1 and 2 have the same affiliation, use the same number “1”, then number 3 becomes number 2.
III. Abstract:
     1. under the methods section, point out the types of health sectors surveyed.
IV. Methods:
    1. Rephrase the statement “An institution-based cross-sectional study was carried out between 30 July and 18 September 2019 using WHO/HAI tools to collect price and availability from pubic and private sectors in Addis Ababa, Ethiopia's capital city.” You have not collected prices and availability. Instead, you have collected data on prices and availability.
    2. Keep 1 table
V. Results:
   1. “As shown in Figure 1, the availability of at least one essential psychotropic medicine from each therapeutic class was only observed only in six public medicine retail outlets, …”. Delete the repeated word “only”.
   2. Some tables can be better replaced by figures.
