# Supplementary material for: Access to essential psychotropic medicines in Addis Ababa: A cross-sectional study
Source: PLoS One. 2023 Jul 14;18(7):e0283348. doi: 10.1371/journal.pone.0283348 (PMC10348529; doi:10.1371/journal.pone.0283348)
Supplement: Attachment — Submitted filename: Point by Point Response 2.docx [file pone.0283348.s006.docx]

Point-by-point response

1. We suggest you thoroughly copyedit your manuscript for language usage, spelling, and grammar. If you do not know anyone who can help you do this, you may wish to consider employing a professional scientific editing service. The name of the colleague or the details of the professional service that edited your manuscript

Now edited as suggested by Prof Kaleab Asres, Editor in Chief of Ethiopian Pharmaceutical Journal
 In the ethics statement in the Methods, you have specified that verbal consent was obtained. Please provide additional details regarding how this consent was documented and witnessed, and state whether this was approved by the IRB.

Included in the revised section

2. We note your current Data Availability Statement: "The data underlying the results is available from the first Author."

Dataset is attached with the revised version and the statement has been modified accordingly.
